# Supplementary material for: Causal Relationship between Aging and Anorexia Nervosa: A White-Matter-Microstructure-Mediated Mendelian Randomization Analysis
Source: Biomedicines. 2024 Aug 16;12(8):1874. doi: 10.3390/biomedicines12081874 (PMC11351342; doi:10.3390/biomedicines12081874)
Supplement: Supplementary file 1 [file biomedicines-12-01874-s001.zip › Supplementary Figure/Figure S4/Singe SNP plot of MR analysis for TL to ubm-b-1579.pdf]

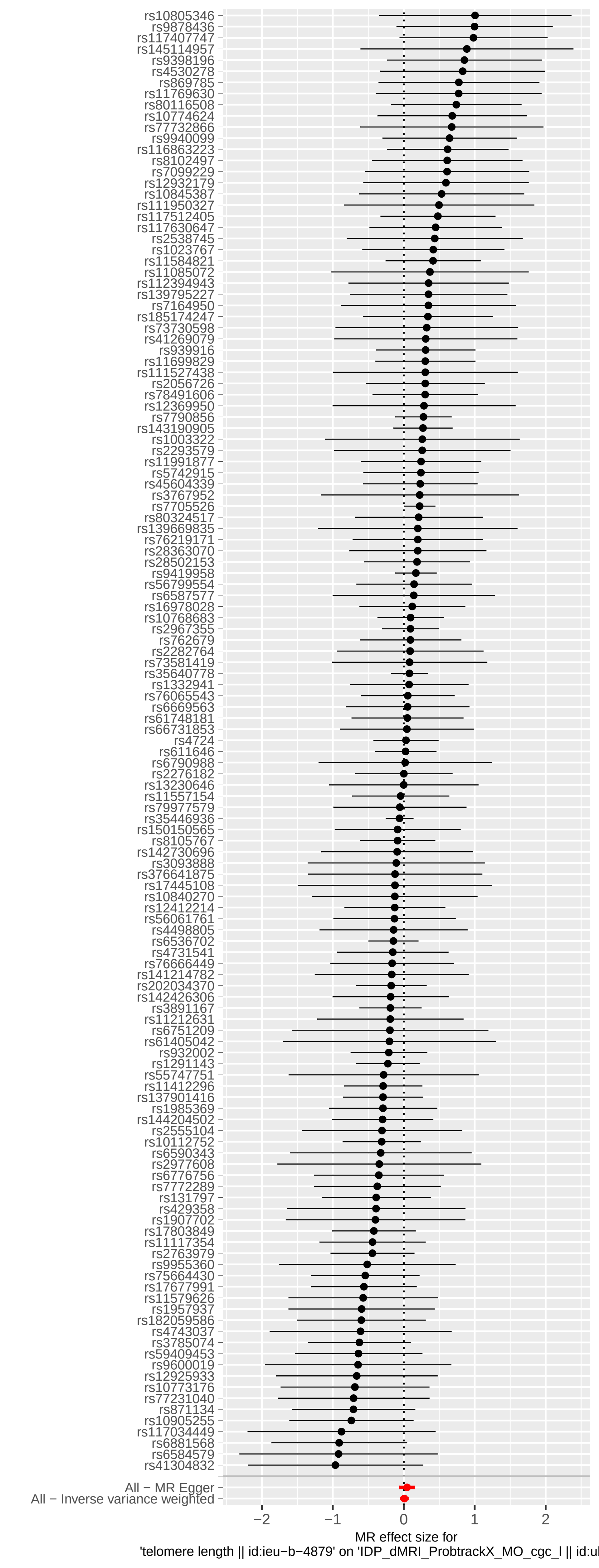
rs10805346
rs9878436
rs117407747
rs145114957
rs9398196
rs4530278
rs869785
rs11769630
rs80116508
rs10774624
rs77732866
rs9940099
rs116863223
rs8102497
rs7099229
rs12932179
rs10845387
rs111950327
rs117512405
rs117630647
rs2538745
rs1023767
rs11584821
rs11085072
rs112394943
rs139795227
rs7164950
rs185174247
rs73730598
rs41269079
rs939916
rs11699829
rs111527438
rs2056726
rs78491606
rs12369950
rs7790856
rs143190905
rs1003322
rs2293579
rs11991877
rs5742915
rs45604339
rs3767952
rs7705526
rs80324517
rs139669835
rs76219171
rs28363070
rs28502153
rs9419958
rs56799554
rs6587577
rs16978028
rs10768683
rs2967355
rs762679
rs2282764
rs73581419
rs35640778
rs1332941
rs76065543
rs6669563
rs61748181
rs66731853
rs4724
rs611646
rs6790988
rs2276182
rs13230646
rs11557154
rs79977579
rs35446936
rs150150565
rs8105767
rs142730696
rs3093888
rs376641875
rs17445108
rs10840270
rs12412214
rs56061761
rs4498805
rs6536702
rs4731541
rs76666449
rs141214782
rs202034370
rs142426306
rs3891167
rs11212631
rs6751209
rs61405042
rs932002
rs1291143
rs55747751
rs11412296
rs137901416
rs1985369
rs144204502
rs2555104
rs10112752
rs6590343
rs2977608
rs6776756
rs7772289
rs131797
rs429358
rs1907702
rs17803849
rs11117354
rs2763979
rs9955360
rs75664430
rs17677991
rs11579626
rs1957937
rs182059586
rs4743037
rs3785074
rs59409453
rs9600019
rs12925933
rs10773176
rs77231040
rs871134
rs10905255
rs117034449
rs6881568
rs6584579
rs41304832
All − MR Egger
All − Inverse variance weighted
−2
−1
0
1
2
MR effect size for
'telomere length || id:ieu−b−4879' on 'IDP_dMRI_ProbtrackX_MO_cgc_l || id:ul
